# Nasopharyngeal carcinoma: a model cancer to understand tumor rejection

**DOI:** 10.1038/s41392-025-02273-0

**Published:** 2025-06-17

**Authors:** Gerard J. Nuovo, Esmerina Tili, Carlo M. Croce

**Affiliations:** 1 GNOME Diagnostics, Powell, OH USA; 2 Department of Cancer Biology, The Ohio State University Comprehensive Cancer Center, and The Ohio State University College of Medicine, Wexner Medical Center, Biomedical Research Tower, Columbus, OH USA

**Keywords:** Translational research

**Dear Editor**,

In a recent study published in *Proc Natl Acad Sci USA*, we demonstrated that the impaired expression of MHC-I due to elevated levels of *miR-155* contributes to immune evasion in diffuse large B cell lymphoma.^1^ This research builds on our previous publication, showing that the silencing of ICOSL by *miR-155* overexpression,^2^ combined with the newly identified role of *miR-155* in regulating MHC-I, are key events in disallowing even large numbers of infiltrating cytotoxic T cells, that we call “frustrated T cells”, from eliminating the tumor cells.

Cancers must evade immune surveillance to proliferate and metastasize. Nasopharyngeal carcinomas (NPCs) present a unique opportunity to study how cancers avoid immune surveillance, particularly given the intense infiltration of cytotoxic CD8+ T cells, a histological marker of the disease.^3^ Most NPCs are classified as WHO types 2 and 3, characterized by poorly differentiated carcinoma cells, and are strongly associated with infection by Epstein–Barr virus (EBV).^3^ Although NPCs are sensitive to radiotherapy and chemotherapy, up to 58% of patients experience recurrent disease. The failure rate of checkpoint inhibitors in NPCs is as high as 70-80%, similar to other cancers. NPCs are strongly positive for PD1/PDL1, however, only 20% seem to respond to immunotherapy.^4^

We examined nine NPCs, each associated with EBV infection and characterized by intense CD8 + T cell infiltrates, for the expression of two EBV-encoded non-coding RNAs (EBER-1 and −2) using in situ hybridization (ISH). In all cases (9/9), a strong signal (3+, i.e., 50% or more target cells positive) for EBV-EBER-1/2 RNAs (green) was evident in basically all carcinoma cells (as shown in panel a) but absent in the surrounding mononuclear cells. These data raised the question: how can the EBV+ tumor cells evade immune elimination despite being surrounded by cytotoxic T cells, whose killing activity renders successful current cancer immunotherapies?

We have published that *miR-155* targets ICOSL, the ligand for Inducible T-cell costimulator (ICOS), consequently impairing the ability of T cells to recognize and eliminate malignant cells.^2^ In addition, we recently showed that *miR-155* indirectly reduces the expression of MHC-I molecules,^1^ that are needed for presenting tumor antigens to CD8 T cells. Furthermore, a report by Dr. Lieberman’s group,^5^ subsequently confirmed by several other publications, has established that EBV induces the expression of *miR-155*. We therefore analyzed NPC tissues for *miR-155* and EBV-RNAs expression using ISH, and for the presence of ICOS, ICOSL, and MHC-I proteins using immunohistochemistry. As evident in panel a, there was a 1:1 correlation between the EBV-RNAs (green) and *miR-155* (blue) as demonstrated by co-expression analyses (yellow), suggesting that only EBV-infected NPC cancer cells expressed *miR-155*. *MiR-155* was not detected in the mononuclear cell infiltrate nor was it present in the adjacent normal epithelia (not shown). Conversely, while the normal adjacent nasopharynx showed strong expression of MHC-I and ICOSL (not shown), the EBER-1/2 NPC cancer cells (green) displayed a near total loss of ICOSL (not shown) and MHC-I (red) (panel b), with however a strong co-expression (yellow) of EBER-1/2 (green) and PDL1 (red) (panel c). Notably, the predominant immune infiltrates, which represent a large portion of each lesion, consisted of T cells expressing ICOS, as indicated by the co-expression (yellow) of ICOS (green) and CD3 (red) in the tumor-associated lymphoid infiltrates (panel d). Thus, the T cells surrounding the carcinoma cells of NPC expressed ICOS whose coupling with ICOSL is essential for tumor destruction.

The key findings of this study are that: (1) The EBV+ carcinoma cells in NPC are the primary source of the markedly elevated levels of *miR-155* as well as PDL1; and (**2**) The *miR-155* upregulation correlates with the loss of both ICOSL and MHC-I expression, which would help render the tumor cells invisible to the intense surrounding T cell infiltrates. We propose that by concurrently inhibiting ICOSL and MHC-I expression, high levels of *miR-155* found in NPC contribute to the immune evasion mechanisms employed by the tumor (Fig. 1).

**Fig. 1:**
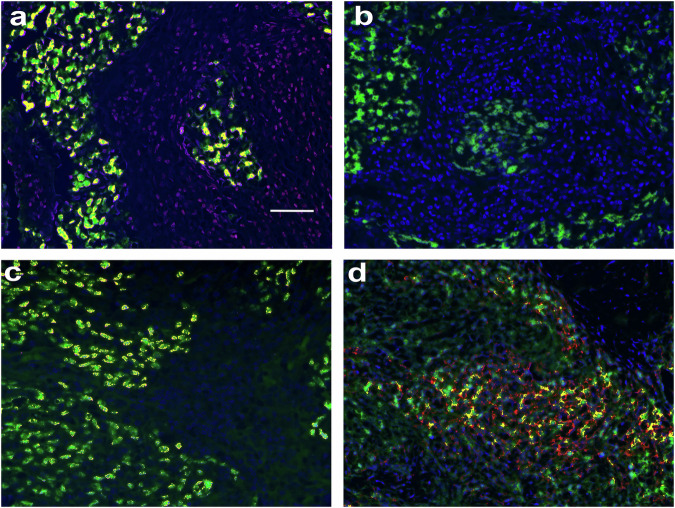
The sustained high levels of miR-155 in EBV+ carcinoma cells lead to the loss of both ICOSL and MHC-I expression, thus impairing the recognition and elimination of tumor cells by activated T cells. a The EBV+ carcinoma cells in NPC show high expression of *miR-155*. Merged image showing that EBER-1/2 positive cells (fluorescent green) strongly co-localize (fluorescent yellow) with *miR-155* (fluorescent blue). **b** The NPC cancer cells directly adjacent to the normal nasopharynx that stained positive for MHC-I (not shown) do not demonstrate either ICOSL (not shown) or MHC-I expression (expected signal fluorescent red) but do contain EBER-1/2 (fluorescent green). **c** The NPC cancer cells do co-express EBER-1 (fluorescent green) and PDL1 (fluorescent red; co-expression seen as fluorescent yellow). **d** Co-localization showing strong co-expression (fluorescent yellow) of the T cell marker CD3 (fluorescent red) and ICOS (fluorescent green) in the tumor infiltrate of an NPC. Counterstain in (**a**) is fast red. Counterstain in (b-d) is hematoxylin. The scale bar is 75 microns

Our study provides novel insights into why the failure rate of checkpoint inhibitors is often as high as 70-80% in NPC.^4^ The cytotoxic T cells in NPC are activated, in the sense of intense proliferation and their abundant expression of ICOS which primes them for tumor eradication. However, the *miR-155-*induced loss of both ICOSL and MHC-I expression by the tumor cells concur to impair T cell killing activity, allowing tumor cells to evade T cell obliteration. As a result, cytotoxic T cells become *frustrated* in their ability to recognize, bind, and eliminate tumor cells. It can be argued that the EBV infection that is at the epicenter of NPCs, has evolved mechanisms that disable the immune system across multiple pathways, including the loss of ICOSL and MHC-I plus the increased expression of PDL1. Otherwise, the cancer cells would have likely been eradicated by the extensive T cell infiltration, impairing virus survival. These findings underscore a new strategy that can be used by cancer cells to frustrate cytotoxic T cells, which, despite infiltrating a tumor en masse, are rendered unable to eradicate it. The role played by *miR-155*, a microRNA normally needed for both antiviral and anti-cancer immune responses, in allowing tumor evasion underscores the challenges posed by developing effective therapies for NPC and highlights the need for therapeutic strategies combining the use of *miR-155*-inhibitors and forced re-expression of ICOSL in tumor cells.

## Supplementary information

Supplemntal material

## Data Availability

All data have been presented in the main text and supplementary materials.
