# Supplementary material for: Nasopharyngeal carcinoma: a model cancer to understand tumor rejection
Source: Signal Transduct Target Ther. 2025 Jun 17;10:187. doi: 10.1038/s41392-025-02273-0 (PMC12170846; doi:10.1038/s41392-025-02273-0)
Supplement: Supplementary file 1 — Supplemntal material [file 41392_2025_2273_MOESM1_ESM.docx]

Supplementary Materials for

Nasopharyngeal carcinoma: a model cancer to understand tumor rejection

Gerard J. Nuovo, Esmerina Tili, Carlo M. Croce

Correspondence to: Carlo.Croce@osumc.edu

**This PDF file includes:**

Materials and Methods

**Materials and Methods**

**Clinical samples and controls**: The patient samples (formalin fixed paraffin embedded biopsy material) originated from the files of Folio Biosciences. Clinical information included the diagnosis and age/sex of patient. There was biopsy material from 9 patients (mean age 55.2 years). This study, based on de-identified formalin fixed, paraffin embedded tissues, was exempt from IRB review. Five of the cases had normal adjacent nasopharynx tissue which, with 5 unremarkable tonsils, served as the negative controls. Four-micron tissue samples were placed on sequentially labeled slides, baked at 60^0^C for 30 min, and stored at RT with an hematoxylin and eosin stain performed to verify the diagnosis.

**Immunohistochemistry (IHC):** IHC was done by Dr. Nuovo. The specific antibodies used (source and catalogue numbers; Ab = ABCAM, Prot = Proteintech) were as follows: CD3 (Ab16669), CD4 (Roche 790-4423), CD20 (Roche 760-2531), ICOSL (Prot 8687), ICOS (Prot 8685), RELA (Ab7970), for MHC-I, two antibodies were tested, Ab22432 and Invitrogen PAS-14413 that gave similar results), PDL1 (Ab205921) and PD1 (Ab237728). All required antigen retrieval for 30 minutes at 95^0^C using an EDTA solution. The IHC protocol used the Leica Bond Max (Buffalo Grove, IL) automated platform; the Fast red (DS9390) and the DAB (DS9800) detection kits were used and gave equivalent results.

***In situ* hybridization (ISH): ISH for *miR-155* was performed by Dr. Nuovo, as previously described in detail in Supplementary material Ref. 1. In brief, the tissue was pretreated for 4 minutes in proteinase K solution (0.1 μg/ml), washed, and then hybridized overnight at 37**^0^**C with an LNA anti-*miR-155* probe digoxigenin tagged at its 5’ end and then detected with an anti-digoxigenin-AP conjugate with NBT/BCIP as the chromogen. ISH for the EBER-1/2 RNA was done with the RNAscope assay as previously described in detail in Supplementary material Ref. 2. In brief, the target sequence was the human herpes 4 isolate SDTW400 EBER-1 and -2 genes, complete sequence from ACD (catalogue # 310271). The assay was done per the manufacturer’s protocol that used DAB as the chromogen.**

**Multispectral and co-expression testing and statistical analyses:** Co-expression experiments were performed by analyzing a given tissue section for one protein using the DAB (brown) chromogen and analyzing the other protein with Fast Red chromogen and, when done with *miR-155*, using NBT/BCIP (blue). Co-expression analyses were done using the Nuance software **as previously described in detail in Supplementary material Ref. 2, Chapter 11**. Quantification for the signal with either single IHC or multi-labeled IHC was done using either the InForm software or manual counting which yielded equivalent results. Statistical analysis was done using the InStat Statistical Analysis Software (version 3.36) and a paired t-test (also referred to as a “repeated measure t-test”). The null hypothesis was rejected if the significance level was below 5%.

**IHC- in situ scoring:** IHC and ISH scoring was done blinded to the target tested. The standard pathology system of 0, 1+, 2+, and 3+ was used as defined in this study by 1-24% target cells positive (1+), 25-49% target cells positive (2+) and 50% or more target cells positive (3+). Six 200X fields are scored for a given target that yields an average value for each data point. For more details see Reference 2 in Supplementary materials.

**References related to supplementary information:**

1. Nuovo, G. J., Elton, T. S., Nana-Sinkam, P., Volinia, S., Croce, C. M., & Schmittgen, T. D. A methodology for the combined in situ analyses of the precursor and mature forms of microRNAs and correlation with their putative targets. *Nature protocols*, **4**, 107–115, (2009).
2. Nuovo, G. J. In Situ Molecular Pathology and Co-Expression Analyses (Second Edition). Book. *Academic Press,* ISBN 9780128206539; **Chapter 4** - The basics of in situ hybridization; **Chapter 5** - The basics of immunohistochemistry; **Chapter 7** - Recent improvements in immunohistochemistry and in situ hybridization; **Chapter 10** - The recommended protocol for immunohistochemistry; **Chapter 11** – Co-expression analyses; (2020).
